# Rheumatoid arthritis is associated with rs17337023 polymorphism and increased serum level of the EGFR protein

**DOI:** 10.1371/journal.pone.0180604

**Published:** 2017-07-11

**Authors:** Chung-Ming Huang, Hsin-Han Chen, Da-Chung Chen, Yu-Chuen Huang, Shih-Ping Liu, Ying-Ju Lin, Yuan-Yen Chang, Hui-Wen Lin, Shih-Yin Chen, Fuu-Jen Tsai

**Affiliations:** 1 School of Chinese Medicine, China Medical University, Taichung, Taiwan; 2 Division of Immunology and Rheumatology, Department of Internal Medicine, China Medical University Hospital, Taichung, Taiwan; 3 Division of Plastic and Reconstructive Surgery, China Medical University Hospital, Taichung, Taiwan; 4 Taiwan LandSeed Hospital, Pingjen City, Taoyuan, Taiwan; 5 Genetics Center, Department of Medical Research, China Medical University Hospital, Taichung, Taiwan; 6 Center for Neuropsychiatry, China Medical University Hospital, Taichung, Taiwan; 7 Department of Microbiology and Immunology, and Institute of Microbiology and Immunology, School of Medicine, Chung Shan Medical University, Taichung, Taiwan; 8 Department of Optometry, Asia University, Taichung, Taiwan; 9 Department of Pediatrics, China Medical University Hospital, Taichung, Taiwan; 10 Department of Medical Genetics, China Medical University Hospital, Taichung, Taiwan; Children's National Health System, UNITED STATES

## Abstract

**Objective:**

We have previously described the association of rheumatoid arthritis (RA) prevalence and two epidermal growth factor receptor (EGFR) SNPs (rs17337023 and rs2227983) among the Taiwanese population. This present study aimed to elucidate whether the SNPs can alter the expression of EGFR in the progression of RA.

**Methods:**

The cohort study included 366 Taiwan’s Han Chinese RA patients and 326 age and gender matched healthy controls. Blood samples collected from the participants were analyzed to determine their serum EGFR levels and to identify EGFR SNPs from their genomic DNA. Genotyping for EGFR SNPs was performed by restriction fragment length polymorphism (RFLP) assay. The relationship between EGFR SNP and the clinical manifestations of RA was evaluated.

**Results:**

Our results showed that a statistically significant difference in genotype frequency distributions at rs17337023 SNP for RA patients and controls (*p* ˂ 0.05). In addition, compared with the haplotype frequencies between case and control groups, the RA patient with the GT haplotype appeared to be a significant “protective” haplotype compared with other haplotypes (OR: 0.73, 95% CI: 0.59–0.91; p = 0.005). Furthermore, the increased serum level of EGFR was also observed in RA patients (*p* ˂ 0.001).

**Conclusion:**

Our study showed that RA is associated with rs17337023 SNP in EGFR gene and increased serum level of the EGFR protein. These findings suggest EGFR is worthy of further investigation as a therapeutic target for RA.

## Introduction

Rheumatoid arthritis (RA) is an autoimmune disease in which the body’s defense system attacks every part of the body, particularly the synovial joints. This can result in a chronic and systemic inflammatory condition and can lead to disability if the patient is not properly treated. Although the exact causes of RA are not fully known, most scientists think that a combination of genetic traits and environmental triggers are responsible for causing the disease. RA affects nearly 1% of the population worldwide [1–3] and seems to affect females three times more likely than men. The prevalence of RA increases with age and is more common among people between 40 to 65 years old of age. In Taiwan, Chinese men and women have overall lower prevalence and incidence rates than many other countries [4,5]. But similar to other countries, RA patients in Taiwan also have higher mortality than the general population, particularly those who suffer from RA-related complications. RA imposes a huge social and economic burden on the national social welfare and healthcare systems [1,5–9]. In advanced cases, patients with joint deformities require not only medical and surgical care, but also rehabilitation and psycho-social support.

Symmetrical swollen synovial joint is a characteristic of RA that arises from leukocyte infiltration, suppressed synovial fluid leukocyte apoptosis and synovial hyperplasia at the sites [10–12]. The epidermal growth factor receptor (EGFR) has a crucial role in aggressive tumor growth [13–16]. Similar to tumor growth, hyperplastic synovium of RA also expresses EGFR and its ligands [17,18]. The accumulated data from experimental animal models resembling RA and the clinical trials suggest that activated synovial fibroblasts, also known as rheumatoid arthritis synovial fibroblasts (RASF), recruit inflammatory leukocytes and induce pannus growth and angiogenesis in the synovial lining of the joints in RA patients [10]. EGFR is activated by binding with its specific ligands, which then undergoes a transition from an inactive monomeric form to an active homodimer, then it induces by auto phosphorylation of tyrosine Y residues in the C-terminal domain and elicits activation of downstream signal transduction cascades leading to DNA synthesis and cell proliferation [19–21]. Gene polymorphism and mutations that lead to EGFR overexpression, over activity, or constant activation has been associated with a number of cancers [13,14,16]. A number of studies demonstrated that the serum EGFR concentration is significantly higher in RA patients than the healthy controls [18,22]. EGFR has been proposed as a valuable therapeutic target for the treatment of joint inflammation in patients with RA. We have previously described the association of rheumatoid arthritis prevalence and two single nucleotide polymorphism sites (SNPs, rs11543848, which has now merged into rs2227983 and rs17337023) of EGFR among Taiwan’s Han Chinese population [23]. This present study we increased the sample size of the cohort and aimed to elucidate whether the SNPs, can alter the expression of EGFR in the progression of RA.

## Materials and methods

### Study population

This study was approved by the institutional review board (IRB) of china medical university hospital (Taichung, Taiwan). Prior to patient enrollment, all participants provided written informed consent. For this study, we enrolled 366 RA patients and 326 healthy subjects from China Medical University Hospital in Taiwan. As described in detail previously [23], section material and methods. Patients with RA were recruited based on the 1987 revised criteria of the America College of Rheumatology [24]. The gender-age-matched unrelated healthy controls from the general population were selected through physical examination. Nephelometry was used to detect rheumatoid factor (RF), values ≧ 20 IU/ml were defined as positive. A presence or history of extra-articular manifestations in patients with RA was recorded [25]. All individuals’ samples were collected by venipuncture for genomic DNA isolation.

### Genomic DNA extraction and genotyping (polymerase chain reaction and restriction enzyme analysis)

Genomic DNA (gDNA) was prepared from peripheral blood and it was according to the standard protocols of the DNA extraction kit (Genomic DNA kit, Roche, USA). Polymerase chain reaction (PCR) was used to identify the EGFR polymorphisms, including rs2227983 and rs17337023. Polymerase chain reaction was carried out with a total volume of 50 μL, containing 50 ng of genomic DNA, 2–6 pmol of each primer, 1× Taq polymerase buffer (1.5 mM MgCl2) and 0.5 units of AmpliTaq DNA polymerase (Perkin Elmer, Foster City, CA, USA). In the study of the EGFR rs2227983 SNP, the primers used were forward-5'-TGCTGTGACCCACTCTGTCT-3' and reverse-5’-CCAGAAGGTTGCACTTGTCC-3'. For the EGFR rs17337023 SNP, the primers used were forward-5'-ATATATGCCAAAGAAGTAG-3' and reverse-5'-TGATCAGGACAGAGGACAG-3'. PCR amplification was performed in a PCR thermal cycler (GeneAmp PCR System 2400, Perkin Elmer). The PCR cycling conditions for EGFR rs2227983 SNP examination were as described in detail previously [23], section material and methods. The EGFR rs2227983 SNP was analyzed by PCR amplification, followed by restriction enzyme analysis with BstNI. The EGFR rs2227983 SNP was categorized as excisable (GG homozygote), non-excisable (AA homozygote) and partially-excisable (AG heterozygote). The PCR cycling conditions for EGFR rs17337023 SNP examination were as described in detail previously [23], section material and methods. The EGFR rs17337023 SNP was analyzed by PCR amplification, followed by restriction enzyme analysis with BsrI. The EGFR rs17337023 SNP was categorized as excisable (TT homozygote), non-excisable (AA homozygote) and partially-excisable (AT heterozygote) [23].

### Haplotype analysis

Haplotypes were inferred from un-phased genotype data using the Bayesian statistical method available in the software program Phase 2.1 [26]. Both of these two SNPs were analyzed with the Phase 2.1 software and the population data were divided into different groups with the presence of haplotypes above 5%.

### Enzyme-linked immunosorbent assay (ELISA) quantitative assay of EGFR

An enzyme-linked immunosorbent assay (ELISA) was developed for the determination of EGFR in human Serum (Cat No. 30–7110, ALPCO Diagnostics, USA). As described in detail previously [27], serum samples were diluted 1/200 in the dilution buffer which was within the range of standard curve, and the human EGFR in the serum samples were bound to monoclonal mouse antibodies against human EGFR, which were immobilized on the surface of the microtiter plates. Then, the quantification of bound human EGFR was carried out by adding a rabbit anti-human EGFR antibody. Detection of the bound rabbit antibody was performed by a peroxidase labeled goat anti-rabbit antibody. The signal was determined by measurement of OD_450_ nm.

### Statistical analysis

Chi-square test will be used to determine statistically significant differences in allele/ genotype frequencies of EGFR SNPs (rs2227983 and rs17337023) between case and control groups. Among the RA patients, genotype groups with different clinical variables were also compared using chi-square test. The results are considered statistically significant when p values are less than 0.05. The Odds ratios (ORs) were calculated from the genotypic frequency and allelic frequency with a 95% confidence intervals (95% CIs) for the EGFR SNPs (rs2227983 and rs17337023). The statistical analysis was performed using SPSS version 11.

## Results

### Genotypic and allelic frequency distributions of two EGFR SNPs among Taiwan’s Han Chinese population

The genotypic and allelic frequency distributions of two SNPs (rs2227983 and rs17337023) in the EGFR gene are summarized in Table 1. The Hardy-Weinberg model was used to describe and predict genotype and allele frequencies in our study cohort. We observed that A allele was the major allele at both EGFR SNPs in the population regardless of whether or not they were in the patient group or the control group; at rs2227983 SNP, the A allele frequencies were 52.5% (384 out of 732) for the patient group and 52.6% (343 out of 652) for control group. While at rs17337023 SNP, the A allele frequencies were 57.8% (409 out of 732) for the patients and 54.1% (346 out of 652) for the controls. By comparing the genotypic distributions between RA patients and healthy controls, our data indicated that individuals with either homozygous A or heterozygous A allele (i.e. AA or AT) at rs17337023 SNP site were at a higher risk for developing RA (p ˂ 0.05).

**Table 1:** Genotypic and allelic frequencies of EGFR genetic polymorphisms in the RA patients and controls.

	RA patients	Controls	OR (95% CI)	*P value* ^*a*^
**EGFR-1808(rs11543848)**	[n = 366 (%)]	[n = 326 (%)]		
GG	76 (20.8)	68 (20.8)	Ref	
AG	196 (53.6)	173 (53.1)	-	
AA	94 (25.7)	85 (26.1)	-	
AA+AG	290 (79.2)	258 (79.1)	1.01(0.7–1.45)	1.9516
Allelic frequency				
Allele G	348 (47.5)	309 (47.4)	Ref	
Allele A	384 (52.5)	343 (52.6)	0.99(0.8–1.23)	1.9120
**EGFR-2133(rs17337023)**	[n = 354 (%)]	[n = 320 (%)]		
TT	43 (12.1)	62 (19.4)	Ref	
AT	213 (60.2)	170 (53.1)	-	
AA	98 (27.7)	88 (27.5)	-	
AA+AT	311 (87.9)	258 (80.6)	1.74(1.14–2.65)	0.0196 *
Allelic frequency				
Allele T	299 (42.2)	294 (45.9)	Ref	
Allele A	409 (57.8)	346 (54.1)	1.16(0.94–1.44)	0.3421

CI, confidence interval; OR, odds ratio.

^a^ *P* value with Bonferroni correction

* Statistically significant

The estimated haplotype frequencies for both rs2227983 and rs17337023 SNP sites were summarized in Table 2. Four different EGFR haplotypes emerged in our study cohort, AA was the most common haplotype observed in both RA patients (42.9%) and the healthy controls (47.1%). We also made a comparison of haplotype frequency distributions between RA patients and healthy controls; our data indicated that individuals with GT haplotype seemed to be protected from developing RA (OR: 0.73, 95% CI: 0.59 to 0.91 and p = 0.005). Nonetheless, individuals with either GA or AT haplotype may be at a higher risk for developing RA (GA haplotype: OR: 2.19, 95% CI: 1.54 to 3.12 and p ˂ 0.001; AT haplotype: OR: 1.87, 95% CI: 1.24 to 2.82 and p ˂ 0.005).

**Table 2:** Distribution of EGFR haplotype frequencies in the RA patients and controls.

Haplotypes ^a^	RA Patients (%)	Controls (%)	OR (95% CI)	*P value* ^*b*^
	(n = 366)	(n = 326)		
AA	42.90%	47.10%	0.84(0.68–1.04)	0.4292
GT	32.90%	40.10%	0.73(0.59–0.91)	0.02 *
GA	14.60%	7.30%	2.19(1.54–3.12)	4.72E-05*
AT	9.60%	5.40%	1.87(1.24–2.82)	0.0128*

CI, confidence interval; OR, odds ratio.

^a^ Order of single nucleotide polymorphisms comprising the EGFR haplotypes: rs2227983 and rs17337023

^b^ *P* value with Bonferroni correction

* Statistically significant

### Increased serum level of EGFR in RA patients

**Fig 1:**
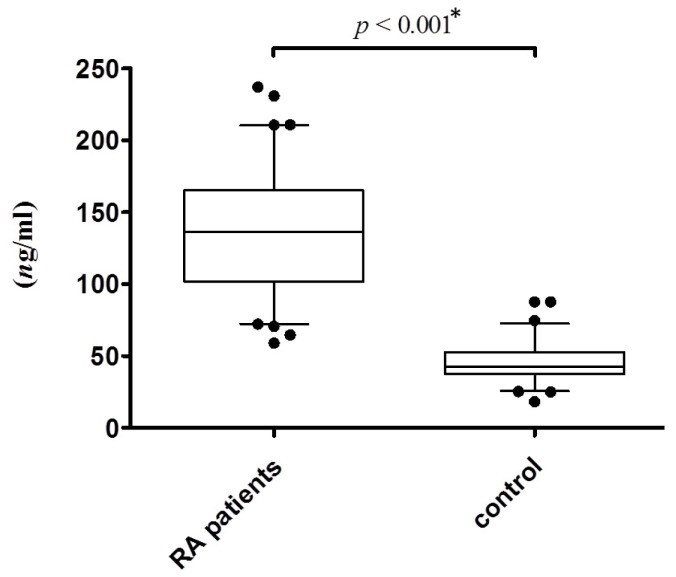
The expression level of EGFR in the serum of RA patients and controls. p value was calculated by T-Test and Mann-Whitney U test. * means data is statistically significant.

Serum levels of EGFR were available from the serum preparations of 80 RA patients and 79 healthy controls. For the remaining patients and controls, insufficient serum was collected to quantify EGFR. There was a significant increase of serum EGFR concentration in RA patients (Fig 1, p ˂ 0.001). The mean EGFR concentrations, per milliliter of serum, in the samples of RA patients and the healthy controls were as follows: 138.1±41.9 ng/mL for RA patients and 46.1±14.8 for healthy controls. RA patients had three folds higher serum EGFR levels than their age-matched, gender-matched and race-matched healthy controls.

### Biochemical and clinical assessments of RA patients carrying risk- and non-risk-associated EGFR SNPs at rs17337023

We examined markers for signs of anemia, kidney dysfunction, abnormal lipid profile and liver disorder. There was no statistically significant difference between the RA-risk-associated group (AA or AT at rs17337023) and RA-non-risk-associated group (TT at rs17337023) in various markers that were evaluated (Table 3). Inflammatory and RA-specific markers included ESR, CRP, RF, and anti-CCP. Although the data did not have any statistical significance, RA patients who carried TT at rs17337023 seemed to be protected against inflammation and were less likely to develop severe RA (Table 3). The mean of ESR, CRP, RF and anti-CCP level in the serum samples of RA-risk-associated patients (AA or AT at rs17337023) and RA-non-risk-associated patients (TT at rs17337023) are as follows: ESR, 31.2±26.3 mm/hr for AA/AT carriers and 29.7±21.7 mm/hr for TT carriers; CRP, 2.4±2.5 mg/dL for AA/AT carriers and 1.0±1.3 mg/dL for TT carriers; RF, 451.0±639.5 U/mL for AA/AT carriers and 225.3±338.6 U/mL for TT carriers; anti-CCP, 114.1±21.1 EU/mL for AA/AT carriers and 6.2 EU/mL for TT carriers.

**Table 3:** Relationships between EGFR SNP (rs17337023) genotype and clinical signs and findings in patients with RA.

		RA patients		T-Test	Normal reference range
		Genotype at rs17337023		*p* value	
		AA+AT—n (mean±SD)	TT—n (mean±SD)		
**Anemia**	Haemoglobin test of CBC	69(12.07±1.69)	20(12.42±1.4)	0.402	Men: 13.8–18.0 g/dL
					Women: 12.1–15.1 g/dL
**Autoimmune disorder**	Platelet count of CBC	69(235631.88±72331.29)	20(248800.0±51468.54)	0.45	150,000–450,000/μL of blood
**Inflammation**	WBC count of CBC	69(6738.55±2455.87)	20(7708.0±226.22)	0.117	Approximately 7000 WBC/μL of blood
	ESR	70(31.17±26.32)	20(29.65±21.72)	0.814	Men >50 years old: Less than 20 mm/hr
					Women >50 years old: Less than 30 mm/hr
	CRP	30(2.40±2.53)	9(1.01±1.29)	0.124	Undetectable or less than 0–1.0 mg/dL
**Kidney disease**	Creatinine	68(0.80±0.29)	20(0.91±0.88)	0.369	Males: 0.6–1.2 mg/dL
					Females: 0.5–1.1 mg/dL
	Protein in urine	69(7.78±38.11)	20(18.3±66.94)	0.37	Approximately 0 to 8 mg/dL
	RBC in urine	69(4.32±5.66)	20(16.9±57.09)	0.337	Less than 4 RBC/HPF
**Lipid disorder**	Cholesterol	33(193.60±31.35)	5(215.2±54.46)	0.203	Less than 200 mg/dL
	Triglycerides	32(110.28±51.47)	5(147.80±114.65)	0.509	Less than 150 mg/dL
**Liver disease**	SGPT	68(25.81±24.22)	19(20.68±7.99)	0.368	Males: 10–40 U/L
					Females: 7–35 U/L
**RA autoantibodies**	RF	47(450.09±639.52)	15(225.30±338.62)	0.199	Less than 40–60 U/mL
	Anti-CCP	2(114.05±21.14)	1(6.2)	0.15	Less than 20 EU/mL

## Discussion

The severity of RA varies greatly from person to person. The exact cause of RA is unknown and is a very active area of global research. We performed a candidate gene study in order to investigate the association of genetic variants in EGFR, with its expression, and the disease severity in RA patients. EGFR was chosen as a candidate gene because EGFR polymorphisms and mutations have been associated with a number of cancers, including anal cancer [28], colorectal cancer [29], glioblastoma [30], and non-small-cell lung cancer [13,16]. RA is characterized by proliferative and invasive synovial fibroblasts in the synovium [3,10,22], which are a population of cells with properties similar to cancer cells. We investigated the association of SNPs tagging EGFR with its serum level and RA severity in a Taiwan’s Han Chinese study cohort.

EGFR is a transmembrane receptor that is a member of the tyrosine kinase superfamily and is activated by binding to a specific ligand, such as EGF. Upon activation by EGF, EGFR transits from an inactive monomer to an active homodimer [31]. EGFR dimerization stimulates its intrinsic tyrosine kinase activity through the auto phosphorylation of tyrosine (Y) residues in the C-terminal domain of EGFR [32]. This auto phosphorylation evokes a downstream signal transduction cascade by an association with several SH2 domain-containing proteins. These downstream signaling proteins include GRB2-SOS complex in the MAPK pathway, PLC-γ1 in the AKT pathway, and VAV in the JNK pathway [33]. These signal transduction pathways are critical cellular events that lead to enhanced DNA synthesis and cell proliferation. Because hyperplasia in RA is similar to a hyperplastic tumor, scientists believe that EGFR has a role to play in RA pathology [17,18]. EGFR is overproduced by cells present in the synovial joints of RA patients. Several recent studies demonstrated that both serum and synovial fluid EGFR concentrations are significantly higher in RA patients than healthy controls [18,22]. Our study also demonstrated that the serum EGFR concentration is significantly higher in RA patients in Taiwan’s Han Chinese population (Fig 1). This is exactly the reason why EGFR has been proposed as a therapeutic target in the treatment of joint inflammation in RA patients.

We have previously examined the association of rheumatoid arthritis prevalence and the two EGFR SNP sites (rs2227983 and rs17337023) among Taiwan’s Han Chinese population [23]. In the present study, the relatively modest sample size that replicated earlier published data of the SNPs of EGFR in RA cohort. Here, we increased the RA and control sample size from 188 to 366 and 128 to 326, respectively and all data analysis were with Bonferroni correction. Our data still indicated that individuals with A carrier genotype at rs17337023 SNP are at higher risk for RA. We also observed that the genotype distributions here with a little different when compared with the previously one, it should be due to the reason of the cohort sample size changed.

SNP-rs2227983 is located in exon 13, while SNP-rs17337023 is located in the intron between exon 14 and exon 15 [13,14]. EGFR is composed of an extracellular ligand (e.g. EGF) binding domain and an intracellular tyrosine kinase domain. There are four different EGFR isoforms: full-length isoform A contains both the extracellular and intracellular domains, while truncated isoform B, C, and D are those lacking in intracellular tyrosine kinase domains. Although they have been shown to diminish cell proliferation in vitro, the role of truncated EGFR isoforms remain unclear. Indeed, these are examples of truncated isoforms that negatively regulate their full-length counterparts [34,35]. Both rs2227983 and rs17337023 are located in the lower portion of the extracellular ligand binding domain (exon 13 to 16), which is adjacent to the transmembrane domain (exon 17), the situation perhaps increasing EGFR activity by increasing full-length EGFR transcript expressions.

In addition, our findings revealed that EGFR rs17337023 AT and TT genotypes were associated with susceptibility to RA. We found that individuals with A carrier alleles were at higher risk for RA. To the best of our knowledge, there is only one report from one other laboratory which looked at the association between EGFR polymorphism and RA [36]. In contrast to our findings, Hashemi et al. reported that EGFR T variant was associated with an increased risk of RA in the Iranian population, and that carriers of T allele were at a 1.56-fold increase in getting RA. In the present study, the minor allele (T allele) frequency of EGFR rs17337023 in RA patients and controls were 0.422 and 0.459, respectively. The frequency of T allele in RA patients (0.729) and controls (0.633) have been reported in Zahedan, Southeast Iran [36].

Here, we not only reporting the characterization of a SNP variation of EGFR in rheumatoid arthritis patients, but also the EGFR protein levels in RA patient’s serum. Our results indicate a significantly higher level of EGFR in patients than in controls. To our knowledge, this is the first report on EGFR serum protein levels in the biggest cohort of RA patients and it is a very important information in RA development.

Both RF and anti-CCP are specific markers for RA because they are produced as part of the process that leads to joint inflammation in rheumatoid arthritis [37]. CRP is usually ordered along with ESR; they measure how much inflammation is in the body [38]. Although ESR and CRP are not specific tests, the flaring up of these values do indicate that you have inflammation somewhere in your body. The RA patients who carry TT at rs17337023 also tended to have lower mean of RF, anti-CCP, ESR, and CRP than the group who carried AT or AA, but no statistical significance difference was observed, possibly due to the small sample size of the subgroup analysis. The sample size is closely tied to statistical power. We have to admit that we have had a small sample size in some subgroups. Actually, it was difficult to get enough blood for both routine and RA-specific blood tests; therefore, additional studies are needed in the future to validate these results by using a larger cohort of RA patients.

In conclusion, our pilot study showed that RA is associated with rs17337023 SNP in EGFR gene and increased serum level of the EGFR protein. These findings suggest EGFR is a valuable therapeutic target in the treatment of RA and thus, is worth further investigation.

## Supporting information

S1 File Supporting information files_EGFR rawdata. The raw data of two SNPs (rs2227983 and rs17337023) in the EGFR gene and the serum levels of EGFR among Taiwan’s Han Chinese population.
(XLSX)
